# Influence of Atmospheric Contaminants on the Work Function of Graphite

**DOI:** 10.1021/acs.langmuir.3c01459

**Published:** 2023-08-15

**Authors:** Ruobing Bai, Nathan L. Tolman, Zhenbo Peng, Haitao Liu

**Affiliations:** † Department of Chemistry, University of Pittsburgh, Pittsburgh, Pennsylvania 15260, United States; ‡ Chemical Engineering College, Ningbo Polytechnic, Ningbo, Zhejiang 315806, P. R. China

## Abstract

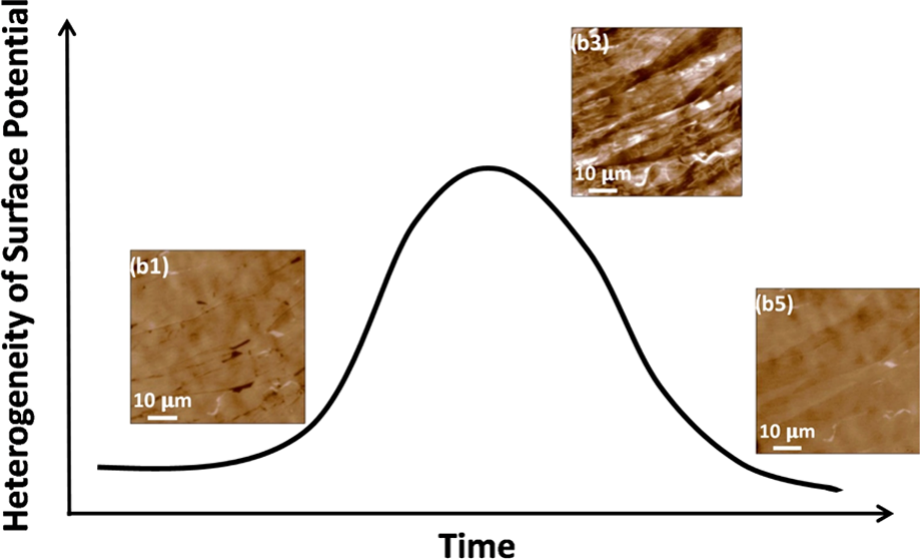

Airborne hydrocarbon contamination occurs rapidly on graphitic surfaces and negatively impact many of their material properties, yet much of the molecular details of the contamination remains unknown. We use Kelvin probe force microscopy (KPFM) to study the time evolution of the surface potential of graphite exposed to ambient. After exfoliation in air, the surface potential of graphite is not homogeneous and contains features that are absent in the topography image. In addition, the heterogeneity of the surface potential images increased in the first few days followed by a decrease at longer exposure times. These observations are strong support of slow conformation change, phase separation, and/or dynamic displacement of the adsorbed airborne contaminants.

## Introduction

Graphitic carbons have excellent physical and electrical properties (e.g., high heat resistance, high heat and electrical conductivity)^1,2^ and these properties make them suitable for a wide range of applications in electrochemistry and energy storage.^3−5^ Graphene, or a single-layer graphite, also has many unique electrical properties and has been extensively researched for potential applications in sensors and electronic devices,^6,7^ such as gas sensors, biosensors, electrochemical sensors, flexible light emitting diodes (LEDs), solar cells and field effect transistors (FETs).^8−13^ These applications all involve surface related processes and hence sensitive to the physical and chemical nature of the graphitic surface.

Work function, being one of the fundamental surface properties, plays a critical role in many interfacial processes such as charge doping, charge separation, and charge injection. Highly oriented pyrolytic graphite (HOPG) is often used as a reference to establish the work function of other materials,^14−16^ because the basal plane of graphite is considered a stable surface in air due to the absence of dangling bonds and surface reconstruction. The work function of graphene depends on the number of layers and was reported to be between 4.46 and 4.64 eV.^14^ However, the work function of HOPG found in the literature varies significantly, even when measured under the same environmental conditions. The reported values range from less than 4.5 eV to as high as 5 eV.^17−21^ Such a wide range of work function values may cause high uncertainty when calibrating the work function of other materials.^22^ The different work function values on HOPG was attributed to surface contamination.^23^ Therefore, when using HOPG to calibrate the work function, the changes in the surface characteristics of the HOPG sample should be considered.

The surface properties of graphite, including work function, are well understood through extensive theoretical and experimental studies.^24−26^ However, in the past decade, we and others have demonstrated, through extensive surface characterizations, such as atomic force microscopy (AFM), water contact angle, Fourier transform infrared spectroscopy (FTIR) and ellipsometry, that the graphitic surface can be contaminated by the trace amount of hydrocarbons in air within a time frame ranging from minutes to days.^27−31^ A recent ultra-violet photoelectron spectroscopy (UPS) experiment showed that after a freshly-exfoliated graphite surface was exposed to air for several days, there was still change in the composition of the contaminants on the surface.^32^ These and other literatures also indicated that the contamination on the graphitic surface may contain hydrocarbon compounds, metal atoms, oxygen and sulfur.^33,34^ A recent study identified, using low-temperature scanning tunneling microscopy (STM) and other measurements, that the contaminants are normal alkanes with lengths of 20–26 carbon atoms when freshly-cleaved samples of two-dimensional (2D) materials were stored in plastic containers.^35^ These long alkanes are likely outgassed from the plastic container; the contaminants from ambient are likely much more complex.

All these findings suggest that the airborne hydrocarbon contaminates are likely one of the sources of inconsistent surface potentials of graphite previously reported in the literature. Therefore, it is of interest to fully understand how this adventitious contamination impacts the surface potential. On the other hand, the time evolution and spatial distribution of the surface potential of graphite can also provide insight into the kinetics of the surface contamination and the spatial distribution of the contaminants. These considerations are the motivations of this study.

There are a number of studies of the surface potential of HOPG using KPFM. Sommerhalter et al. reported that the work function of air-cleaved HOPG is 400 meV lower than that of cleaved in ultra-high vacuum and attributed the difference to adsorbates, although the nature of the adsorbates was not discussed.^20^ Martinez-Martin and co-workers reported the adsorption of polycyclic aromatic hydrocarbons on HOPG and its impact on the surface potential of HOPG. Their experiment was conducted in a vacuum chamber and the source of the aromatic hydrocarbon was not determined.^36^ Lee et al. showed that the step edge of graphite creates a contrast in the KPFM image and that such contrast is different for exposed and covered (i.e., step edge underneath a graphene layer) step edges. These work echoes the literature on the self-assembled monolayer structure on graphite^37,38^ and MoS_2_,^39^ clearly indicating that surface potential imaging can reveal the presence, polarity, and phase separation of adsorbed molecules on graphite.

Here, we use KPFM to study the influence of ambient air contaminations on the surface potential of HOPG. We track the changes in surface potential map at the same area of HOPG over several days and report both short- and long-term changes due to the adventitious contamination of HOPG surface by airborne hydrocarbons. Our data revealed complex behaviors in the time evolution of graphite surface during air exposure, previously undetected by conventional AFM imaging.

## Experimental Section

### Materials

HOPG (SPI-2, 10 mm × 10 mm × 2 mm in size) was purchased from SPI Supplies. Scotch brand tape (3 M, Inc) was used to peel off the top layers of the basal plane of the HOPG. Care was made to ensure a complete exfoliation of the HOPG sample. The exfoliated sample was immediately used in the KPFM characterization which typically takes less than 10 min to setup. Note that this study focuses on the long-term (i.e., several days) time evolution of the HOPG surface, therefore, the short delay between the exfoliation and the first KPFM imaging is negligible. The calibration slide used for surface potential calibration was purchased from Motic Microscopes.

### AFM Measurements

Throughout the study, surface morphologies of the samples were characterized using an Asylum MFP-3D atomic force microscope by tapping mode in air with Budget Sensors Tap 190E-G tips (190 kHz, 48 N/m). The lab is housed in a chemistry research building with air conditioning. All experiments were conducted at room temperature. Images were analyzed by Igor Software (version 6.3.7.2) equipped with Asylum Research package. KPFM measurements were performed in amplitude-modulated mode by using a two-pass technique. The first pass was to determine the topography of the surface and the second pass was to measure the contact potential difference of the sample surface in a line-by-line fashion. The applied tip voltage was 3 V. All images were flattened to center data and remove tilt. Due to the flattening, the center of the potential map scale is close to 0 V. Note that flattening the image does not alter the contact potential difference, which is the focus of our data analysis.

## Results and Discussion

Previous studies by us and others have extensively characterized the spontaneous airborne contamination of HOPG and 2D materials. Relevant to this study, ellipsometry data showed that upon exposure to ambient, *a* ∼ 0.5 nm (roughly a monolayer) thick of contaminant layer is formed on the surface of HOPG within 30 min.^30^ The growth of this contaminant layer is accompanied by an increase of the water contact angle, from around ca. 64 to 90°.^28,30^ The FTIR spectrum of the surface showed −CH_2_– vibration peaks after exposure to ambient, indicating the presence of hydrocarbons.^30^ UPS data indicated that the composition of the contaminant changes over time, even after weeks of air exposure.^32^ Conductive AFM characterization showed a much-reduced conductivity between the tip and the HOPG, consistent with the formation of a non-conductive hydrocarbon coating on the surface of HOPG.^40,41^ It is interesting to note that although many of these studies did not control the local atmosphere, surprisingly they reported an overall consistent behavior of HOPG upon air exposure, suggesting at least some of the chemicals responsible for the surface contamination is commonly found across the globe.

**Figure 1:**
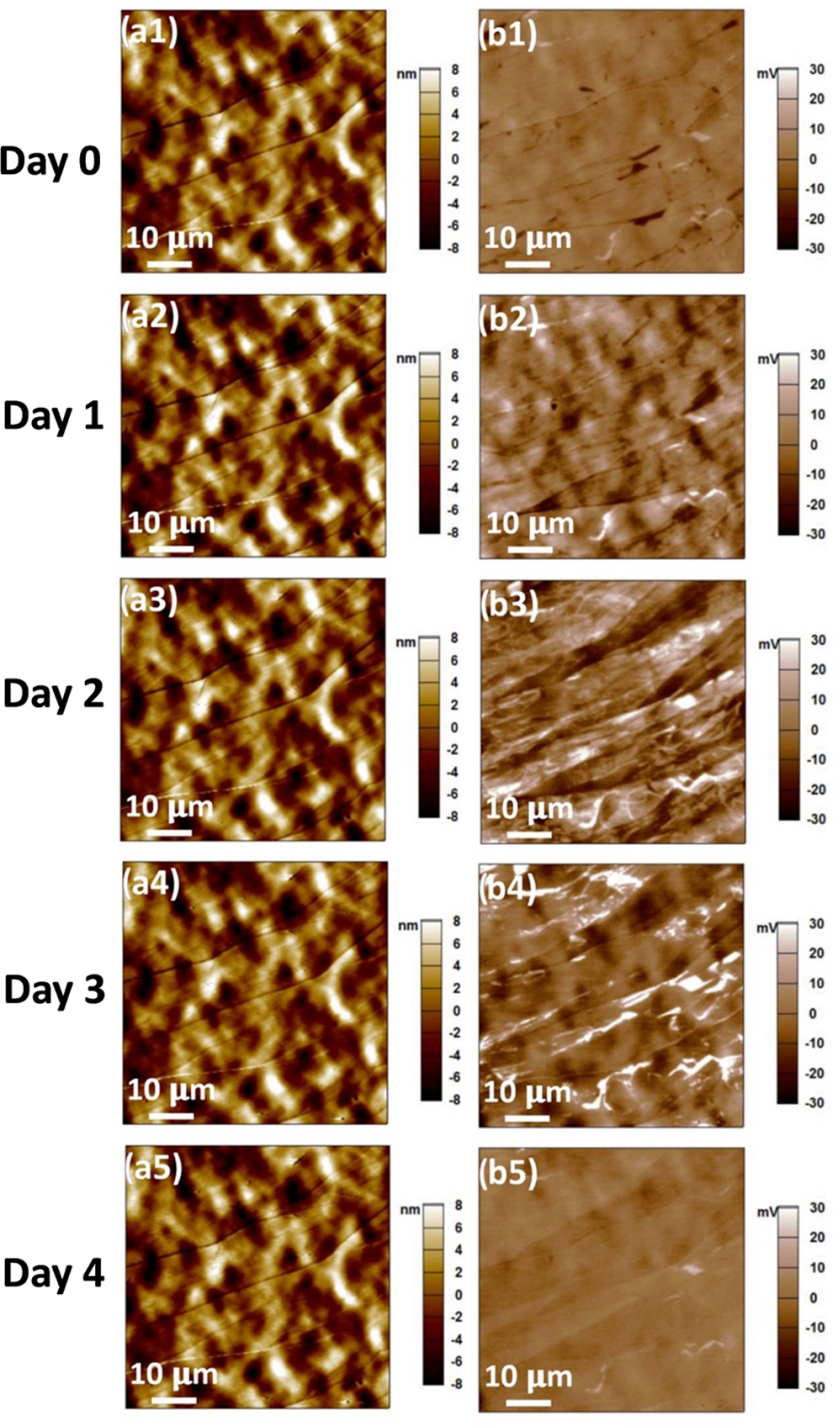
KPFM images of HOPG on different days at the same location. (a1–a5) Amplitude images. (b1–b5) Potential maps, corresponding to different days. Day 0 sample refers to the freshly-exfoliated HOPG (typically imaged within 10 min of exfoliation).

We created a clean HOPG surface by using tape to remove the top layers of HOPG and mounted the sample on the AFM instrument located in a shared facility instrument room. We used KFPM to characterize an HOPG surface at the same location after its cleavage and during its storage in ambient for up to 4 days. The result is shown in Figures 1 and S1. The most important observation here is that while there is no obvious change in the amplitude images (Figure 1a1–a5) collected on different days, the surface potential maps showed significant variations.

On the surface potential map of freshly-cleaved HOPG, we observed micrometer-sized domains, with both positive and negative potential contrasts (Figure 1b1). While many of these domains are not linked to any features in the corresponding amplitude image, some do spatially correlate with step edges. In the latter case, the domains are mostly showing a negative surface potential contrast. In contrast, many of the positive surface potential contrasts (light area) do not correlate with any topography feature. After 1 day of air exposure, the potential map becomes more heterogeneous (Figure 1b2), with a large number of new domain structure appeared. This trend continued on the second day (Figure 1b3) of air exposure, with a noticeable increase in the heterogeneity of the overall image. On the third day (Figure 1b4) and especially the fourth day (Figure 1b5) of air exposure, the surface potential images continue to show significant change; however, the background becomes much more homogeneous. In particular, from day 3 to day 4, many domain features disappeared in the surface potential map. During this 4 day period, the amplitude images are practically the same.

We also tracked the topography and surface potential images of another HOPG sample for up to 14 days (Supporting Figure S2). This sample showed a similar behavior during day 0–5 as we discussed above. After day 5, the surface potential map of the sample remained largely unchanged.

**Figure 2:**
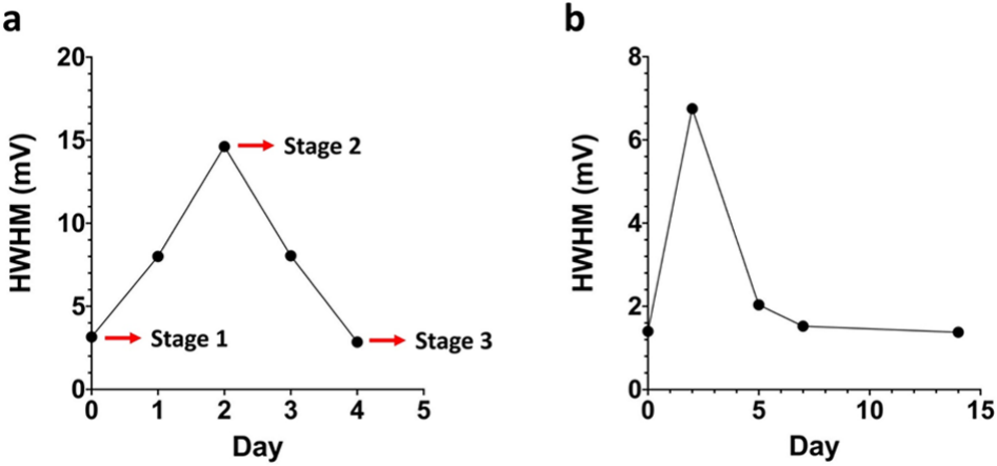
Half width at half maximum (HWHM) for the histogram of the potential map on different days. (a) A 4-day study of the sample data shown in Figure 1. (b) A 14-day study of the sample data shown in Figure S2.

To quantify the change of the surface potential map, the histograms of the contact potential difference (CPD) extracted from the potential map are given in Figures S1 and S2. All histograms show a single peak, which corresponds to the CPD of the background. We also plotted the half width at half maximum (HWHM) of the histogram peaks of the two samples (one sample for 4 days and the other sample for 14 days) as a function of air exposure time in Figure 2. A larger HWHM corresponds to a broader distribution of the CPD, i.e., more light and dark colored features on the KPFM image. For both samples, the HWHM value gradually increased in the first several days and then decreased over time.

**Figure 3:**
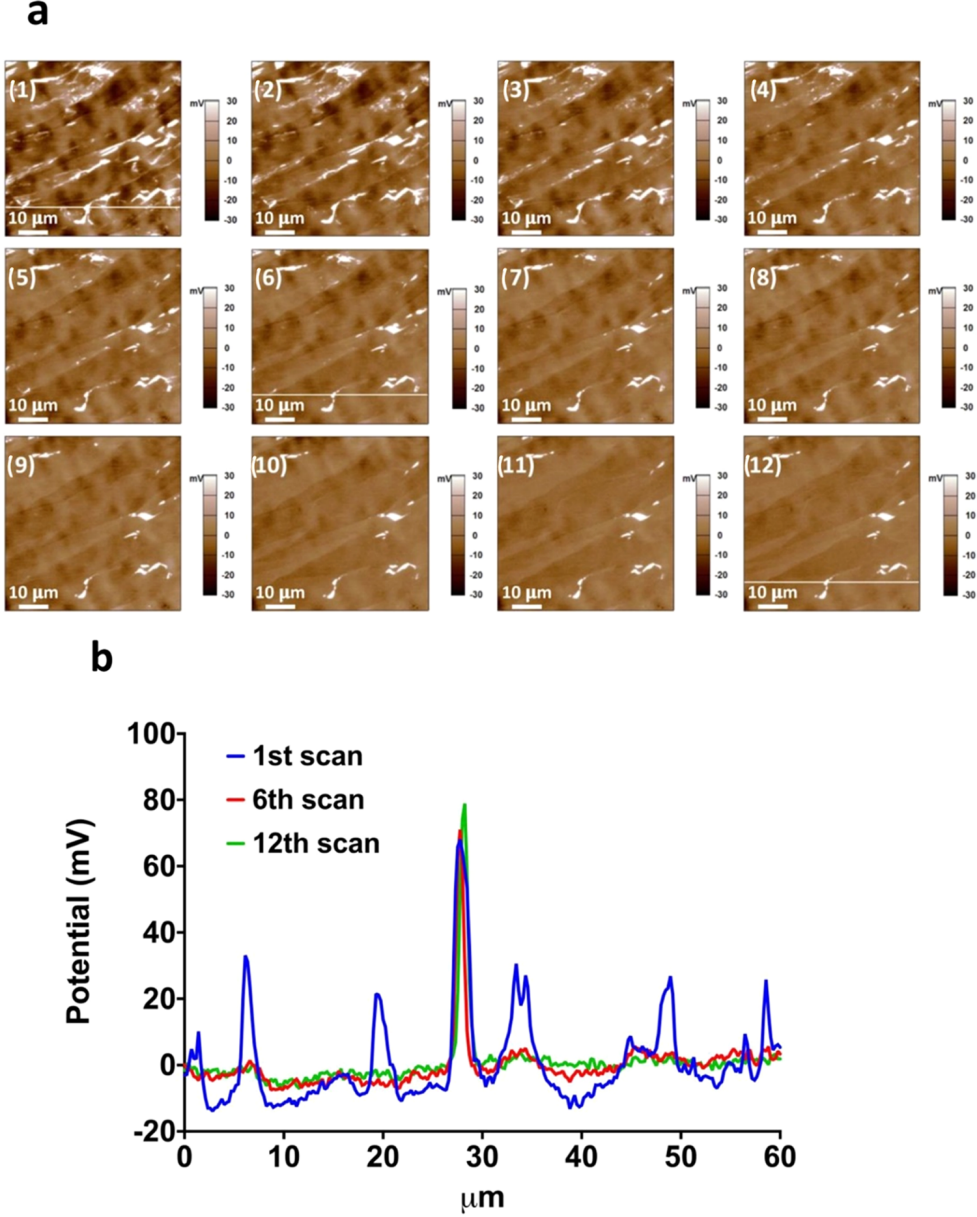
KPFM potential maps of HOPG scanning continuously at the same location on day 3. (a) First to twelfth scan. White cross sections indicate locations where the potential values were measured. (b) Potential values measured at locations where white lines indicate.

In addition to time, the surface potential map of HOPG is also sensitive to the history of AFM scan. For the sample shown in Figure 1, we have carried out continuous scans at the same location during each day. On the 3rd day we found that the potential map showed a gradual change after multiple scans. Figure 3a shows 12 KPFM images sequentially collected on this sample on day 3. Both the positive and negative contrast regions reduced in area after repeated scanning. It can be seen that the potential map stabilized after the 10th scan. To highlight the change in the sample, we plot the cross sections of the potential maps at the same location for the first, sixth, and twelfth scans. Shown in Figure 3b, many peaks in the cross section completely disappeared after repeated AFM scans while one peak remained at the same intensity.

**Figure 4:**
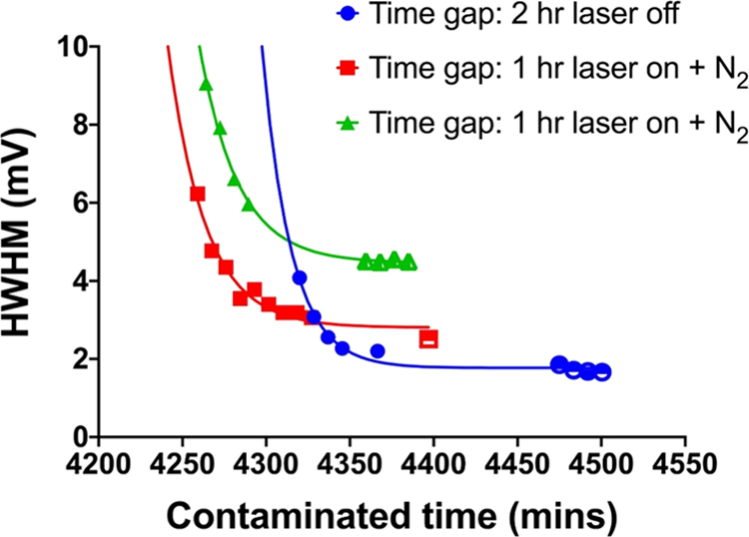
HWHM of the histograms of the potential maps of three HOPG samples after three days of air exposure. The gap between the two clusters of data points is the time delay for each sample. Lines are guide to the eyes.

To verify whether the behavior shown in Figure 3 is caused by AFM laser or humidity, we conducted experiments on the surfaces of other samples that had been contaminated by air for 3 days. We first collected multiple consecutive KPFM images at the same location on the three sample surfaces (each AFM scan takes 8.5 min). These images showed slight differences, and the size of some positive CPD areas gradually decreased (similar to the observation in Figure 3) but did not completely disappear. Then, we stopped the AFM scanning of these samples. For one sample, we turned off the AFM laser for 2 h; for the other two samples, we kept the AFM laser turned on and reduced the humidity in the AFM enclosure with nitrogen flushing for 1 h. After the corresponding processing time was over, we performed KPFM scanning at the same area of the three samples. For comparison, we plotted the HWHM of the histogram of the potential maps in Figure 4. We can see that the HWHM of the three samples dropped sharply at the beginning and finally reached a stable level. The fact that all three samples did not show any recovery of the HWHM indicates that the cause for the change in the potential map during scans is likely time or mechanical contact between the AFM tip and the sample, rather than laser illumination or environmental humidity.

**Figure 5:**
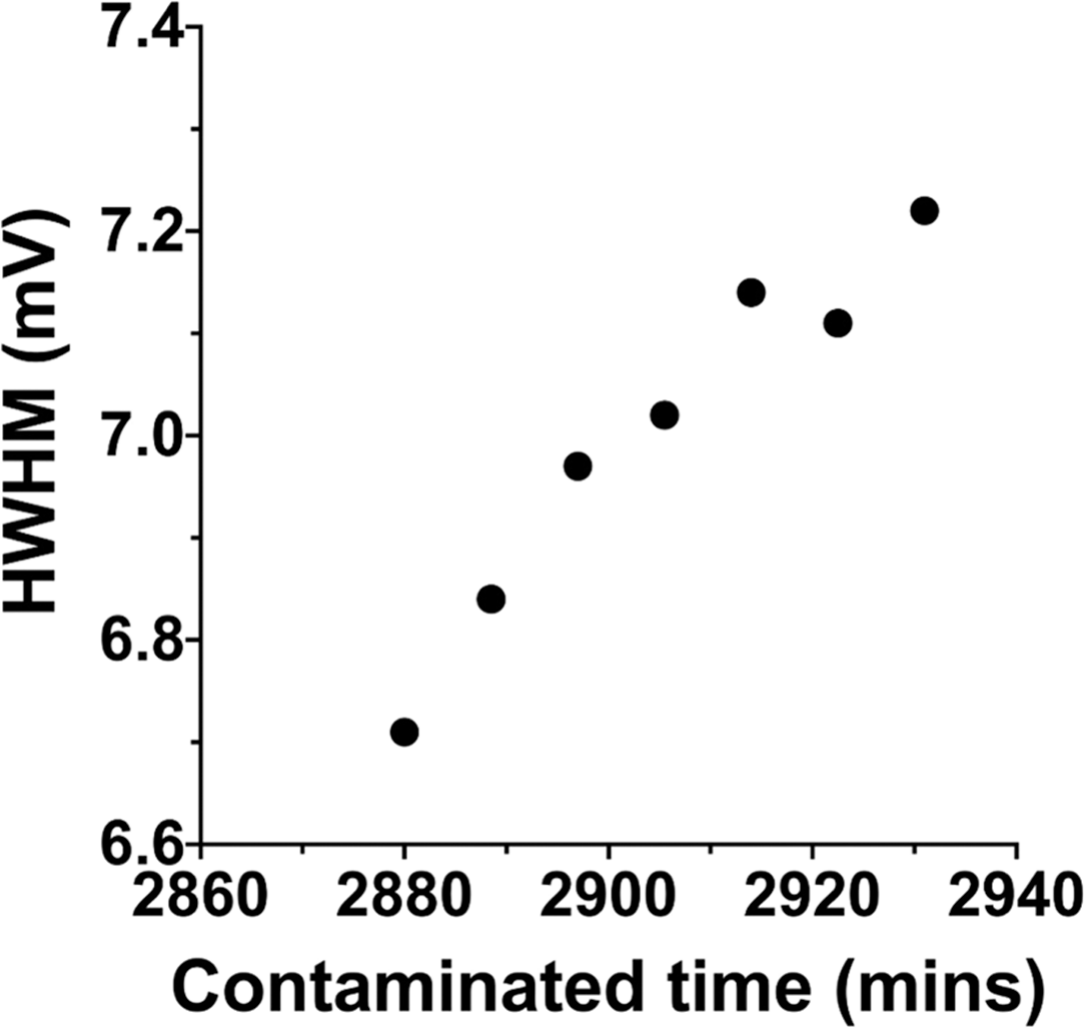
HWHM of the histogram of the potential maps of one sample after 2 days of air exposure.

Intriguingly, for another sample on day 2 of air exposure, we also found that the HWHM of its potential map gradually increased with the number of KPFM scans (Figure 5). Here, the trend of HWHM change over time is opposite to that on day 3. Noticing that the overall trend of HWHM change is a gradual increase peaking at day 2, followed by decrease, the data again suggest that time is the most significant factor in determining the evolution of the KPFM images.

To analyze our data, it is important to know how the positive/negative CPD features in the potential map is translated to real surface potentials, i.e., is positive CPD features due to a higher or lower surface potential? To answer this question, we used a glass calibration slide with deposited chromium thin film for KPFM scanning. The work function of chromium is about 4.5 eV, and the work function of glass is about 4.7 eV.^42,43^ When imaged with KFPM, we found that the CPD of the chromium metal is larger than that of the background glass, i.e., the area with a larger work function will exhibit a lower CPD in our potential map. Based on the data analysis, we deduce that the work function of the lower CPD region in the HOPG potential map will be larger than that of graphite.

**Figure 6:**
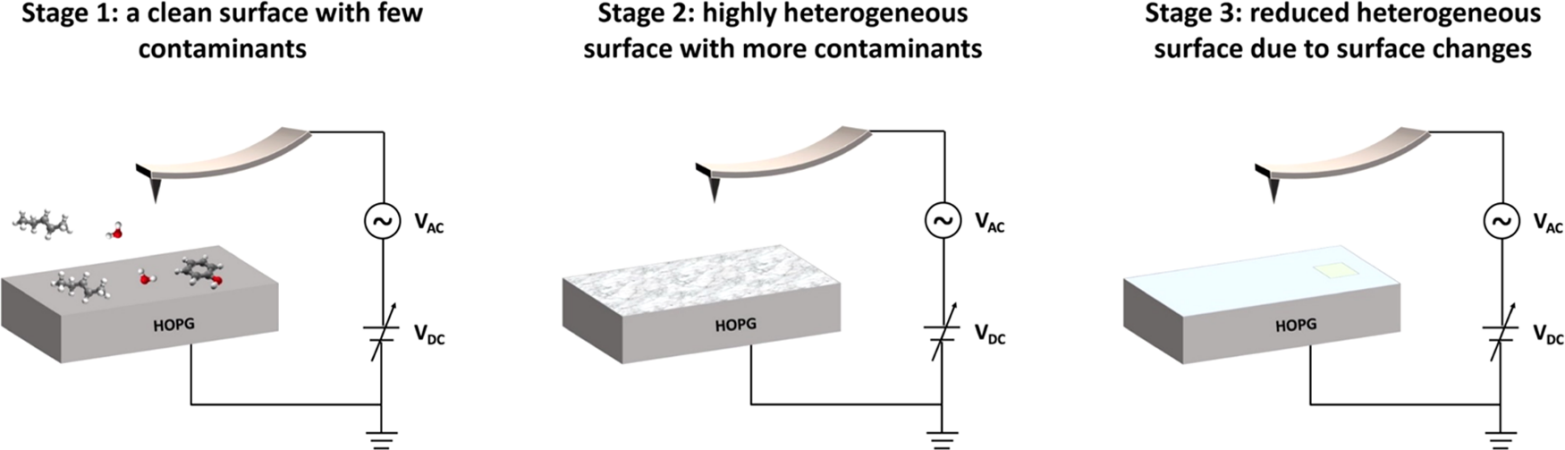
Dynamics of air contaminants on HOPG and their impact on the surface potential.

The data we presented above suggests a dynamic surface where the surface coverage, type, and/or orientation of the airborne contaminants are changing constantly at the HOPG-air interface. Time has the most significant impact on the coverage and type of the contaminant. We describe the influence of contaminants on the surface potential map of the sample in three stages (Figure 2a and Figure 6):

Stage 1 (day 0–1), when the HOPG surface was just exfoliated and the surface coverage of contaminants is low, the surface potential of the sample is mostly uniform as it reflects the nature of clean HOPG or contamination by limited types of hydrocarbons.

Stage 2 (day 2), when more contaminants are absorbed, either due to an increase in their surface coverage or exchange with existing contaminants, the surface potential is highly heterogeneous, due to the different type and/or orientation of the contaminants.

Stage 3 (day 3 and beyond), at even longer air exposure times, we observed a decrease in the HWHM of the distribution of CPD, suggesting that the composition and/or orientation of the contaminants on the sample surface becomes more uniform. The potential map did not show significant change from day 5 to day 12, indicating that equilibrium may have been achieved at day 5 of air exposure.

**Figure 7:**
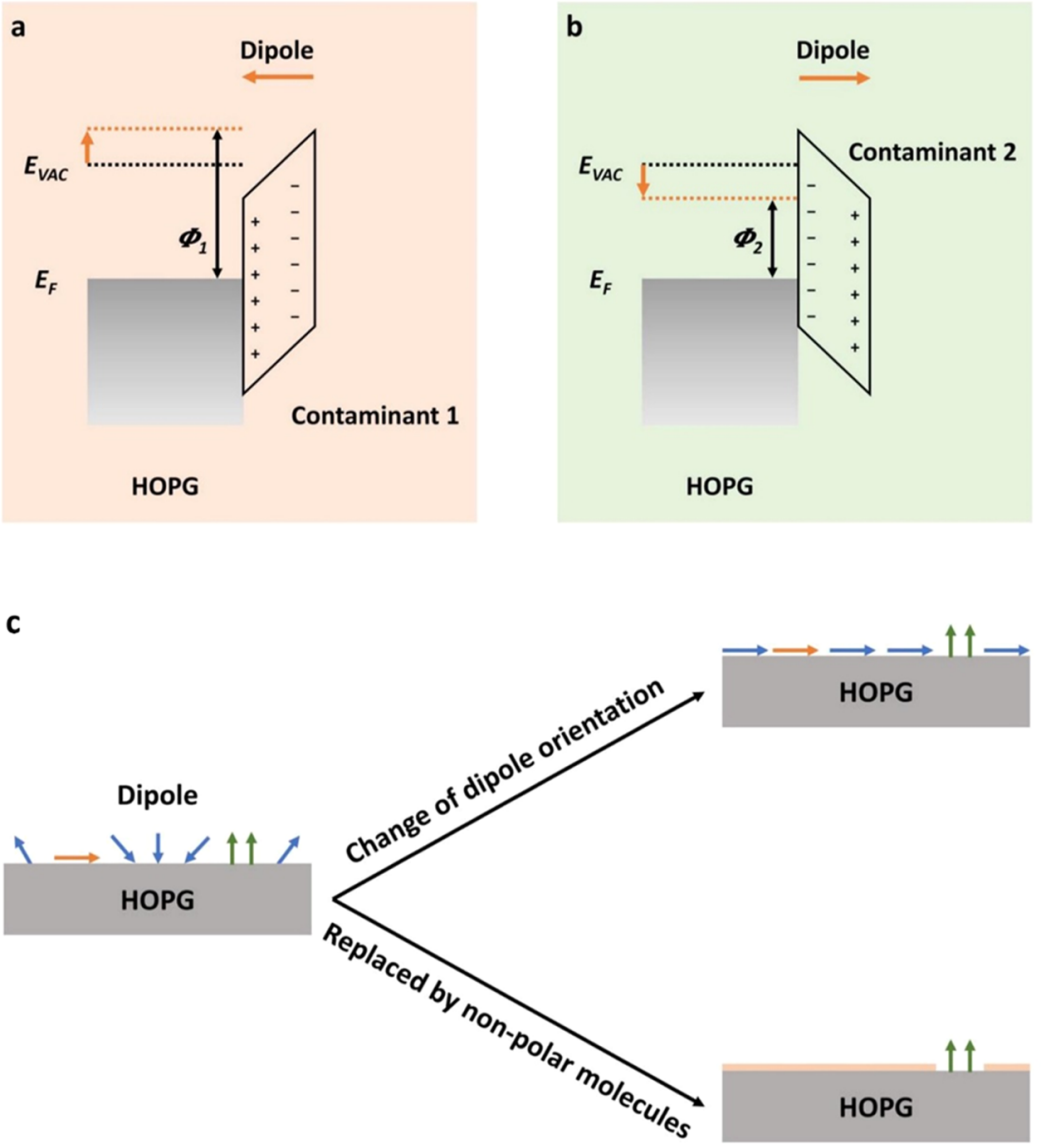
Schematic diagram of: (a, b) the influence of airborne contaminants on the work function of the HOPG surface; (c) possible mechanism for the reduction of heterogeneity of the surface potential.

From the calibration, we know that patches of bright spots (i.e., areas of high CPD) are of lower work function compared to the background. The changes in the CPD features are consistent with a reorganization of the surface adsorbed dipolar molecules. Specifically, the larger work function may be related to the specific dipole direction (from air to HOPG) of some contaminants in that region, because these contaminants will shift the vacuum energy level upwards, thereby increasing the working function of HOPG (Figure 7a).^44,45^ Similarly, the higher CPD (lower work function) area in the HOPG potential map may be related to the presence of some contaminants with the opposite dipole direction (from HOPG to air) in that region (Figure 7b).^46^ Over time, the orientation of the contaminant may change (Figure 7c). We note that overall, the area of bright spots (i.e., high CPD and lower work function) is reduced at long exposure time. If this observation were entirely due to reorientation of dipoles, it would suggest that graphite surfaces favors dipoles oriented from air to HOPG, as having been observed in the case of H_2_O adsorption.^47^

Another possibility for the disappearance of the surface potential contrast in some areas of HOPG is the replacement by other contaminations. These new contaminants may be less polar than the existing ones, or they may shield the potential on the sample surface.^48−52^ Our earlier work, using UPS, showed that the surface contaminant of HOPG undergo continuous change of composition over several weeks.^32^ However, that study only measures the change in the average composition and not their special distribution. To fully uncover the molecular mechanism associated with our observation will require a surface sensitive, structural sensitive and spatially resolved characterization method to image the surface. Possible candidates include secondary ion mass spectrometry (SIMS), surface enhanced Raman spectroscopy, or infrared spectroscopy.^53,54^

## Conclusions

In summary, we identified a strong influence of airborne contaminants on the surface potential map of HOPG surface. The heterogeneity of the surface potential first increased after the exfoliation of HOPG in air, then decreased after ca. 3 days of air exposure. We attribute this time evolution to changes in the coverage, type, and/or orientation of the airborne contaminants on the HOPG surface. We hope that our findings will contribute to a better understanding of the surface properties of HOPG in ambient and in particular the dynamics of its spontaneous contamination by airborne hydrocarbons.

## Supporting Information Available

Histogram of the CPD of the KPFM data in Figure 1 (Figure S1), and time evolution of the KPFM images of an HOPG sample (Figure S2) (PDF).
