# Supplementary material for: Influence of Atmospheric Contaminants on the Work Function of Graphite
Source: Langmuir. 2023 Aug 15;39(34):12159–65. doi: 10.1021/acs.langmuir.3c01459 (PMC10469443; doi:10.1021/acs.langmuir.3c01459)
Supplement: Supplementary file 1 — la3c01459_si_001.pdf [file la3c01459_si_001.pdf]

Influence of Atmospheric Contaminants on the Work Function of Graphite

Ruobing Bai,[1] Nathan L. Tolman,[1] Zhenbo Peng,[2] Haitao Liu[1*]

[1] Department of Chemistry, University of Pittsburgh, Pittsburgh, PA 15260, USA

[2] Chemical Engineering College, Ningbo Polytechnic, Ningbo, Zhejiang, 315806, P. R. China

*E-mail: hliu@pitt.edu

Supplementary Information

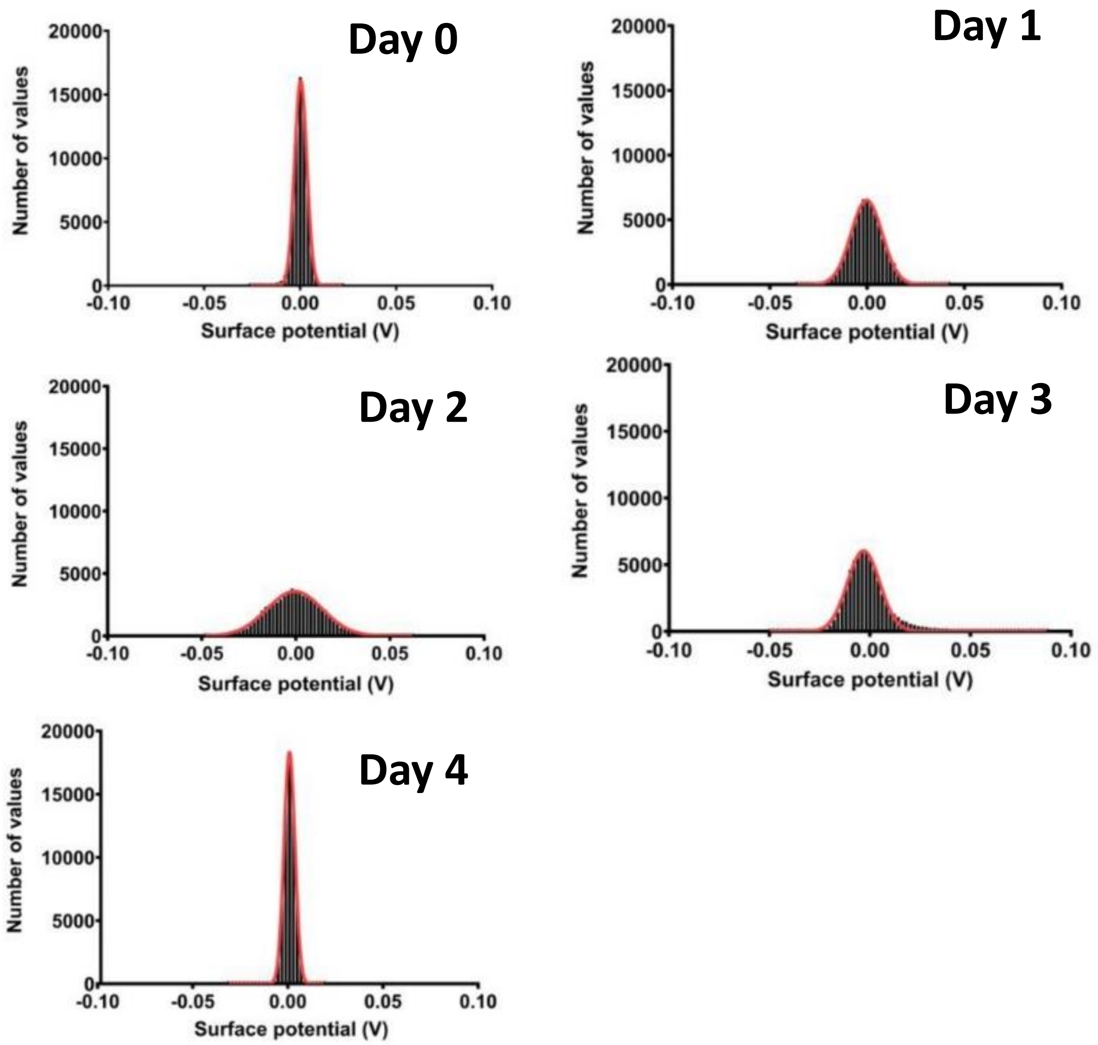

Figure S1. Histogram of the KPFM images of HOPG shown in **Figure 1**.

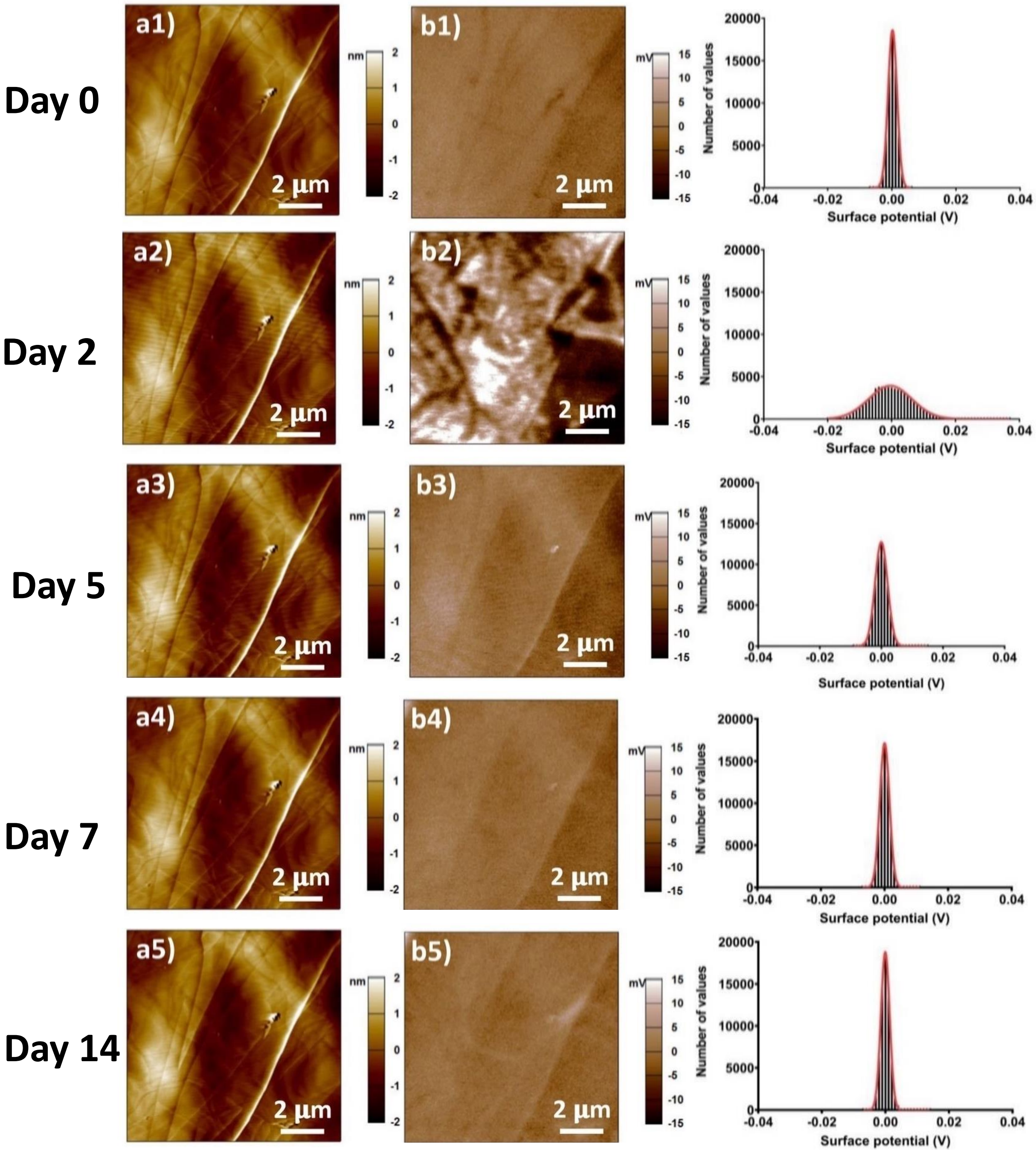

Figure S2. KPFM images of HOPG on different days at the same location. This sample is different from that shown in **Figure 1**. **a1-a5,** Amplitude images. **b1-b5,** Potential maps. **Right column:** Histogram of the respective potential images.
